# Bilateral iatrogenic fracture of the jaw during the removal of third molars: Rare case report

**DOI:** 10.4317/jced.61484

**Published:** 2024-05-01

**Authors:** Emerson-Filipe-de Carvalho Nogueira, Priscila-Lins Aguiar, Thalles-Moreira Suassuna, Fábio-Andrey-da Costa Araújo

**Affiliations:** 1 DDS, MSc, PhD – Specialist in Oral and Maxillofacial Surgery, Department of Oral and Maxillofacial Surgery, University Federal of Pernambuco, Recife, PE, Brazil; 2 DDS, MSc – Specialist in Oral and Maxillofacial Surgery, Department of Oral and Maxillofacial Surgery, University of Pernambuco, Recife, PE, Brazil; 3 DDS, MSc, PhD - Specialist in Oral and Maxillofacial Surgery, Department of Oral and Maxillofacial Surgery, Oswaldo Cruz University Hospital, University of Pernambuco, Recife, PE, Brazil

## Abstract

Mandibular third molar (M3) removal is a routine surgical procedure that can generate a variety of complications, one of the rarest of which is mandibular fracture. The aim of this paper is to report a rare case of bilateral fracture of the jaw during the extraction of mandibular third molars. A 42-year-old woman complained of mandibular pain and malocclusion after the removal of the M3s. The physical examination revealed bone crepitation mandibular bilaterally. Computed tomography confirmed bilateral fracture of the jaw. The patient was submitted to the reduction and fixation of the fractures under general anesthesia, followed by the improvement in the postoperative period. Bilateral mandibular fracture resulting from M3 extractions is a rare occurrence that may be caused by a lack of surgical skill and a flawed diagnosis. The proper, immediate correction could lead to the interruption of the procedure at the time of the first fracture, thereby avoiding the contralateral fracture.

** Key words:**Molar surgery, mandibular fracture, complication.

## Introduction

Mandibular third molar (M3) removal is a common surgical procedure performed by dentists that can result in a variety of complications, such as bleeding, infection, trismus, pain, paresthesia of the inferior alveolar nerve, alveolitis and even mandibular fracture (1). The latter complication is severe and rare, with a reported incidence of less than 1% of cases (1,2).

Flaws in the preoperative assessment and planning, the improper handling of instruments, incorrect ostectomies and tooth sectioning as well as the application of excessive force can cause iatrogenic fractures (1,3). Some intrinsic and anatomic characteristics of the patient can also be considered predisposing factors for the occurrence of this type of complication, such as the degree of bone impaction, tooth and root morphology, bone resilience, the presence of local diseases, infection, age, sex, excessive muscle strength and parafunctional habits (4).

The occurrence of bilateral fracture is an uncommon event when compared to the incidence of unilateral fractures. This is considered an extremely rare complication, especially when resulting from tooth extractions (5). The aim of this paper is to report a clinical case of the occurrence of this complication that is unprecedented in the literature.

## Case Report

A 42-year-old woman visited the emergency ward of a hospital with complaints of intense pain as well as difficulty speaking and chewing after a dental procedure at a clinic approximately five days earlier. The patient reported episodes of recurring pericoronitis related to the mandibular third molars, for which she was submitted to the procedure for the removal of teeth 38 and 48. The patient reported that even under local anesthesia, she felt considerable pain during the extractions and perceived the inability to make contact between the maxillary and mandibular anterior teeth at the end of the procedure. When asking about the malocclusion in the immediate postoperative period, the dentist informed her that it was an expected event explained by the presence of the edema and pain, which are common due to the extensiveness of the surgery performed.

However, even with the use of the prescribed medications and following the postoperative orientations, the inability to close the mouth completely was not resolved. Moreover, the pain and edema increased progressively, leading the patient to seek urgent care at a hospital.

The physical examination revealed diffuse bilateral edema in the lower third of the face, malocclusion with anterior open bite and bone crepitation in the mandibular angle during manipulation, which was accompanied by pain. No scars were found in the area of the extracted teeth, suggesting the absence of incisions for the extractions.

**Figure 1:**
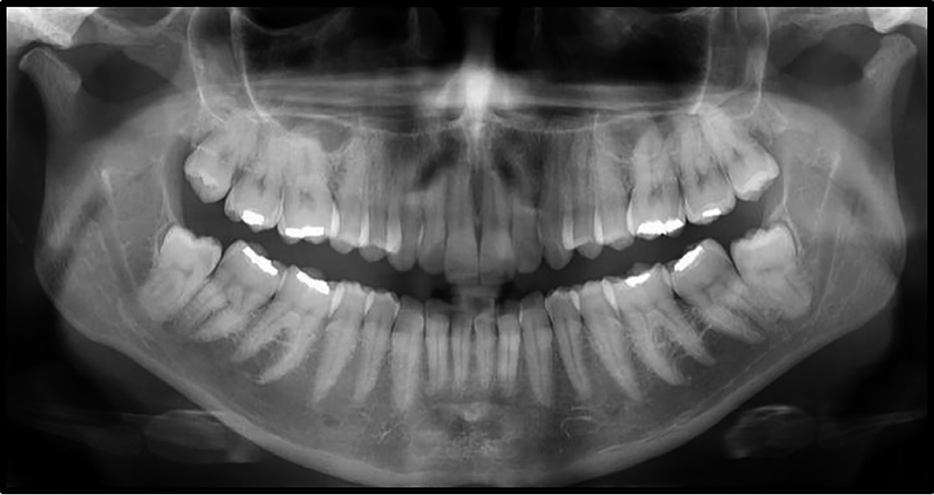
Panoramic radiograph prior to procedure for removal of teeth 38 and 48 presented by the patient in the emergency ward.

The initial radiograph prior to the trauma demonstrated the presence of impacted third molars with long, retentive roots, little space of the periodontal ligament and both exhibiting Pell and Gregory class IIB distoangular positioning (Fig. 1).

**Figure 2:**
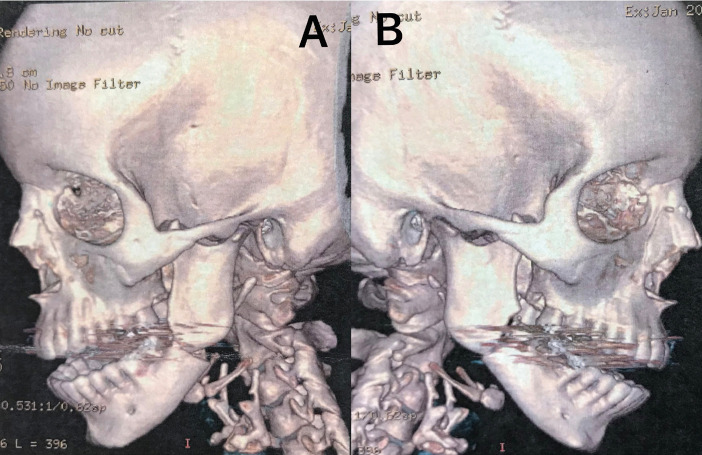
Computed tomogram in 3D reconstruction demonstrating fracture of angle involving the post-extraction area. A: Fracture of left angle. B: Fracture of right angle.

Computed tomography was performed for the complementary diagnosis, which revealed traces of fracture in the mandibular angles bilaterally (Fig. 2).

**Figure 3:**
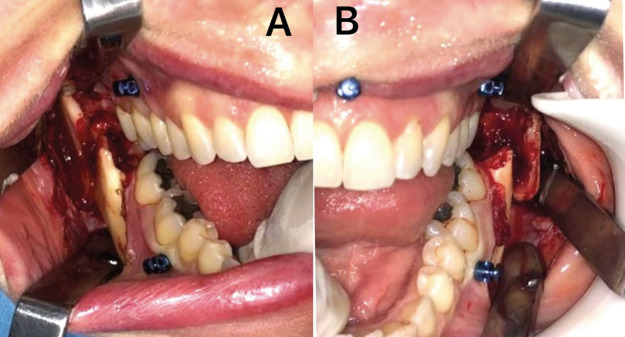
Intraoperative view demonstrating factures and absence of signs of osteotomy in region of removed teeth. A: Right side. B: Left side.

After the diagnosis and preparation, the patient was submitted to surgical treatment under general anesthesia. Intraoral access (Fig. 3) was used for the reduction and fixation of the fractures employing two titanium miniplates on each fractured side. The fixation of the plates and screws near the basilar region of the jaw was assisted with the use of a trocar, followed by suturing with 4-0 monocryl thread. In the postoperative period, an antibiotic and non-steroidal anti-inflammatory drug were prescribed, along with topical cleaning with 0.12% chlorhexidine.

**Figure 4:**
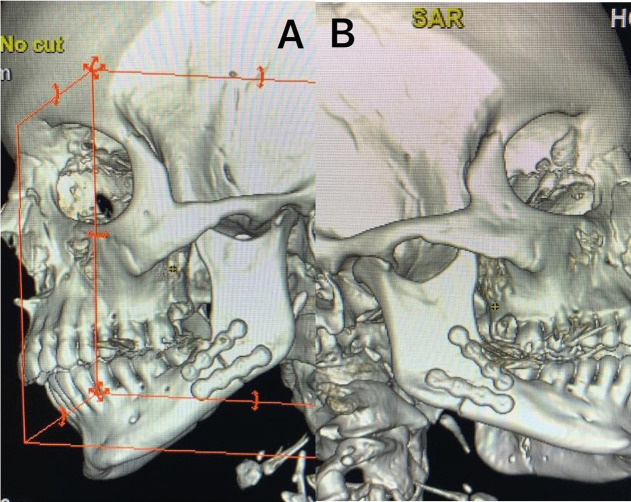
Postoperative computed tomogram demonstrating reduction and fixation fracture with good bone alinement. A: Left side. B: Right side.

The patient was followed up in the postoperative period, with the reestablishment of the occlusion and the return of mandibular functions with no abnormalities. The follow-up image demonstrated the good reduction and fixation of the fractures (Fig. 4).

## Discussion

Mandibular fractures resulting from a tooth extraction can occur during surgery or, more commonly, in the postoperative period of up to four weeks (1,6). The incidence of such fractures ranges from 0.0033% to 0.0075% of cases (1,2) and with a higher incidence in the male sex due to the greater chewing force (1,7,8).

Bilateral fractures are also uncommon and accounted for about 1% of the overall incidence of mandibular fractures. However, the fractures that make up this statistic have facial trauma as the etiology and the literature offers no previous cases of bilateral fracture resulting from tooth extractions (9-12).

M3 extraction is performed more often in patients between 18 and 25 years of age, which is the ideal age range. However, the group most affected by fractures resulting from surgery is between 36 and 60 years of age (1,7,8).

A more advanced age has factors that can contribute to the occurrence of complications, such as diminished bone elasticity, a reduction in the space of the periodontal ligament with potential ankylosis, a higher incidence of bone disorders, such as osteoporosis and atrophy, greater use of medications and slow healing (1,7,8).

In the case reported, the patient was 42 years of age (outside the ideal range for the procedure), which may have contributed to the occurrence of the fractures. Her sex likely did not exert an influence, as the event was independent of chewing force, having occurred during the intraoperative period.

Besides aspects inherent to age, other factors also exert an influence on the incidence of iatrogenic mandibular fractures, such as the magnitude of the impaction, tooth angulation, the length of the roots and the presence of conditions such as infections, cysts and tumors (6,7).

Statistically, mesio-angled and vertical M3s are more associated with iatrogenic fractures, possibly due to the fact that such M3s are more prevalent in the general population (1,13).The dental relationship with the ascending ramus of the type II and III mandible and B and C Pell & Gregory depths also pose an increased risk of fracture, likely due to the greater extraction difficulty by occupying more bone area and requiring greater effort for their removal (13,14).

Despite the predisposing factors, the main factor that contributes to the occurrence of iatrogenic fractures is the inexperience of the surgeon (1,5).

The initial panoramic radiographic of the patient of this report (Fig. 1) revealed M3s in a distoangular and IIB position, which should serve as a predictor of the surgical complexity and exert an influence on the planning and execution of the surgery to avoid complications. Moreover, the patient did not have any comorbidity or made use of any medications that could negatively affect bone strength.

Besides this, necessary surgical techniques, such as the creation of a flap, ostectomy and tooth sectioning, were not performed on either side, likely due to the inexperience of the surgeon, leading to extractions dependent on uncontrolled lever force and resulting in the fractures.

It is also noteworthy that the iatrogenic event occurred bilaterally. This only happened because the surgeon did not perform an immediate diagnosis after the fracture on the first side and repeated the procedure in the same way on the other side, once again revealing a lack of preparedness for the situation.

In cases of tooth impaction in frail jaws, it is important to perform a moderate ostectomy with sectioning of the posterior part of the crown to diminish the force necessary to loosen and extract the M3. Minimal bone removal with greater dependence on the sectioning of the tooth and gentle elevation using minimal pressure should be the standard technique for the removal of third molars with surgical complexity (7,8).

## Conclusions

Mandibular fracture during the extraction of a third molar is considered an uncommon accident. The report of bilateral mandibular fracture due to the performance of a dental procedure has no precedents in the current literature. This type of complication can be prevented with the proper diagnosis, planning and execution, which are factors that require adequate training. Cases such as that reported here should be conducted by a specialist trained in the field of oral and maxillofacial surgery to ensure function and esthetics with minimal sequelae.

## Data Availability

The datasets used and/or analyzed during the current study are available from the corresponding author.
